# Telemedicine for sustainable postoperative follow-up: a prospective pilot study evaluating the hybrid life-cycle assessment approach to carbon footprint analysis

**DOI:** 10.3389/fsurg.2024.1300625

**Published:** 2024-03-18

**Authors:** Ross Lathan, Louise Hitchman, Josephine Walshaw, Bharadhwaj Ravindhran, Daniel Carradice, George Smith, Ian Chetter, Marina Yiasemidou

**Affiliations:** ^1^ Academic Vascular Surgical Unit, Hull University Teaching Hospital NHS Trust, Hull, United Kingdom; ^2^ Centre for Clinical Sciences, Hull York Medical School, Hull, United Kingdom; ^3^ Department of Health Sciences, University of York, York, United Kingdom; ^4^ The Royal London, Barts Health NHS Trust, London, United Kingdom

**Keywords:** telemedicine, sustainability, surgical site infection, surveillance, carbon emissions

## Abstract

**Introduction:**

Surgical site infections (SSI) are the most common healthcare-associated infections; however, access to healthcare services, lack of patient awareness of signs, and inadequate wound surveillance can limit timely diagnosis. Telemedicine as a method for remote postoperative follow-up has been shown to improve healthcare efficiency without compromising clinical outcomes. Furthermore, telemedicine would reduce the carbon footprint of the National Health Service (NHS) through minimising patient travel, a significant contributor of carbon dioxide equivalent (CO_2_e) emissions. Adopting innovative approaches, such as telemedicine, could aid in the NHS Net-Zero target by 2045. This study aimed to provide a comprehensive analysis of the feasibility and sustainability of telemedicine postoperative follow-up for remote diagnosis of SSI.

**Methods:**

Patients who underwent a lower limb vascular procedure were reviewed remotely at 30 days following the surgery, with a combined outcome measure (photographs and Bluebelle Wound Healing Questionnaire). A hybrid life-cycle assessment approach to carbon footprint analysis was used. The kilograms of carbon dioxide equivalent (kgCO_2_e) associated with remote methods were mapped prospectively. A simple outpatient clinic review, i.e., no further investigations or management required, was modelled for comparison. The Department of Environment, Food, and Rural Affairs (DEFRA) conversion factors plus healthcare specific sources were used to ascertain kgCO_2_e. Patient postcodes were applied to conversion factors based upon mode of travel to calculate kgCO_2_e for patient travel. Total and median (interquartile range) carbon emissions saved were presented for both patients with and without SSI.

**Results:**

Altogether 31 patients (M:F 2.4, ±11.7 years) were included. The median return distance for patient travel was 42.5 (7.2–58.7) km. Median reduction in emissions using remote follow-up was 41.2 (24.5–80.3) kgCO_2_e per patient (*P* < 0.001). The carbon offsetting value of remote follow-up is planting one tree for every 6.9 patients. Total carbon footprint of face-to-face follow-up was 2,895.3 kgCO_2_e, compared with 1,301.3 kgCO_2_e when using a remote-first approach (*P* < 0.001). Carbon emissions due to participants without SSI were 700.2 kgCO_2_e by the clinical method and 28.8 kgCO_2_e from the remote follow-up.

**Discussion:**

This model shows that the hybrid life-cycle assessment approach is achievable and reproducible. Implementation of an asynchronous digital follow-up model is effective in substantially reducing the carbon footprint of a tertiary vascular surgical centre. Further work is needed to corroborate these findings on a larger scale, quantify the impact of telemedicine on patient's quality of life, and incorporate kgCO_2_e into the cost analysis of potential SSI monitoring strategies.

## Introduction

Surgical site infections (SSI) pose a significant disease burden globally, complicating 5%–20% of operations (1, 2). With the move towards earlier patient discharge in the current healthcare landscape, the majority of SSI occur after discharge (1). Early diagnosis and treatment of SSI are essential to reduce associated morbidity and mortality; however, access to healthcare services, lack of patient awareness of signs, and inadequate wound surveillance may limit timely diagnosis (3).

Telemedicine has emerged as an innovative method for monitoring patients remotely using electronic communication and information technologies (4). In 2021–2022, 22.9% of the 95.5 million attended outpatient appointments were classified as telemedicine, a substantial increase from 4.3% the previous year (5, 6). Telemedicine is becoming increasingly common in postoperative surgical care and has been shown to improve patient care through the reduction of time to diagnosis and patient travel, without compromising clinical outcomes (7, 8). Furthermore, evidence suggests that telemedicine is highly specific for the diagnosis of SSI and could be utilised as an effective screening tool (9).

In 2019, the National Health Service (NHS)contributed to around 25 million tonnes of carbon dioxide equivalent (CO_2_e) emissions equating to around 7% of the total UK carbon footprint that year (10). A significant contributor of CO_2_e emissions is patient travel, which has almost doubled since 1990 (10). For the NHS to achieve the Net-Zero carbon emission service target by 2045 through the Greener NHS campaign (11), the NHS must adopt innovative approaches to patient care and minimise unnecessary patient travel.

This pilot study aims to evaluate a novel methodology for mapping carbon footprint reduction when modelling remote-first approaches to postoperative follow-up.

## Methods

### Study design

This pilot cohort study mapped carbon emissions of patients followed-up remotely at 30 days after lower limb vascular surgery and modelled clinic carbon footprint for comparative impact assessment. The International Standard Organisation (ISO) 14060:2006 standards for quantification and reporting of greenhouse gases (GHG) were followed (12). All participants provided written consent as part of an ongoing randomised controlled trial (NCT02992951). Ethical approval for this trial was obtained (16/LO/2135) from London–Harrow Research Ethics Committee, and study conduct was in accordance with the Declaration of Helsinki (1975) (13).

The participants were recruited between 6 September 2022 and 1 December 2022, in a tertiary vascular centre in the UK. The eligibility criteria followed those set in the ongoing trial (NCT02992951), which included patients undergoing lower limb vascular surgery closed by primary intention with capacity. Those on antibiotics for conditions not related to their index procedure or had used an investigational device on operative site within four weeks were ineligible for inclusion. Patients were eligible for inclusion even if they did not own a smartphone to transfer wound images. In this instance, relatives, carers, or community nursing teams provided data with patient consent.

### Outcomes

#### Primary outcome

Median reduction in kilograms of carbon equivalent (kgCO_2_e) emissions per patient.

#### Secondary outcomes

- Total metric tonnes of carbon dioxide equivalent (mtCO_2_e) emissions avoided using remote-first postoperative follow-up.
- Median reduction in kgCO_2_e emissions by participants diagnosed with SSI.
- Median reduction in kgCO_2_e by participants without SSI.
- Total distance (km) saved.

### Data collection

#### Process analysis and life-cycle inventory analysis

Participants submitted wound images and completed the Bluebelle Wound Healing Questionnaire (WHQ) at 30 days following surgery, or earlier if a wound-related problem was identified (14). The submitted wound images and Bluebelle questionnaires were reviewed by a trained medical practitioner with experience in diagnosing SSI after vascular procedures. Carbon emissions (kgCO_2_e) for the remote review were mapped based on healthcare resource use of the participants in addition to those incurred due to surgical site infection (such as antibiotic prescription). The patients who developed wound-related problems sought medical advice and treatment through the standard care pathway. Participants also attended face-to-face wound review in comparison with remote review, which occurred on the same day as the remote assessment. Additional healthcare resource use data were collected on general practitioner face-to-face and remote reviews, community and general practice nurse review, antibiotic prescription, blood tests, microbiological sampling, further radiological investigations, and surgical intervention. As the purpose of this study was to model environmental emissions inclusive of postoperative follow-up, kgCO_2_e for the initial admission and initial index procedure were not included within this analysis. The potential kgCO_2_e savings for utilising a telemedicine-first approach were calculated by subtracting the model clinic emissions from the remote emissions.

Carbon emissions were mapped using a hybrid life-cycle assessment approach addressing environmental impact from both bottom-up (prospective item process analysis) and top-down (using a national economic approach to input–output analysis) directions. Utilising a bottom-up approach yields maximum accuracy although it requires physical mapping of individual resources and hence is labour and cost intensive, whereas top-down modelling encompasses system-wide factors beyond the scope of bottom-up assessment. All items are weighed using Model Scout Pro (SPU123) Electronic Balance for items ≤120 g and Marsden medical weighing scales (DS-673SS) for items >120 g.

**Figure 1:**
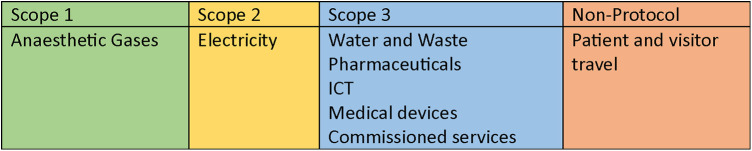
NHS carbon footprint plus evaluated emissions by GHG protocol scopes.

The footprint analysis covers GHG emissions under Scopes 1–3 of the Greenhouse Gas Protocol (15), in addition to personal travel emissions not usually covered within these analyses, providing a comprehensive NHS Carbon Footprint Plus model (16) (Figure 1).

#### Scope 1

Data on anaesthetic gases were collected prospectively through a combination of operative time and anaesthetic agent applied with emissions factors provided by the Sustainable Development Unit (17) and Association of Anaesthetists Anaesthetic Gas Calculator (18). Emissions factors for surgical interventions were applied to operative time providing CO_2_ equivalent for reoperation. No fossil fuels or NHS fleet vehicles were accounted for in this analysis.

#### Scope 2

The Department for Environment, Food, and Rural Affairs (DEFRA) and Business, Energy, and Industrial Strategy (BEIS) GHG conversion factors were applied to the data collected on electricity to provide kgCO_2_e within this scope (19). Electricity data accounted for lighting in both remote and clinic models, and for personal computer use.

#### Scope 3

DEFRA/BEIS GHG conversion factors for water use in addition to waste incineration factors were integrated with resource data providing emissions mapping. Water data collected accounted for handwashing in clinic models (19, 20). The NHS supply chain online catalogue provided individual clinic item costings (21). The National Institute of Health Research (NIHR) interactive costing tool for investigation and intervention tariff provided radiological investigation costs (22). No business travel or metered dose inhaler emissions were utilised within this study.

Pharmaceutical data comprised antibiotics prescribed for SSI. The British National Formulary (BNF) pricing information provided cost information for medications used (23). Medication costs were multiplied by accompanying emissions factors for pharmaceutical data. For oral medications, empty blister packs were weighed and quantified before mapping incineration factors. Intravenous antibiotic packaging were weighed and quantified before applying incineration factors.

National tariff data from the Personal Social Services Research Unit (PSSRU) provided hourly cost data for hospital clinician, general practitioner, and community nurse time (24). For remote review, time to complete assessment was applied to clinician cost and staff services emissions factor. For clinic review, allotted appointment time was multiplied by cost and the staff services emissions factor.

No additional medical devices, freight transport, business services, construction, food and catering, commissioned services, manufacturing, or commuting services’ emissions were utilised or calculated within this study.

#### Emissions outside GHG protocol scope

Patient travel emissions were evaluated by collecting mode of transport, return mileage from home postcode to clinic postcode, and application of the emissions factor for method of transport (DEFRA/BEIS conversion factors) (19).

### Statistics

Data were collected and entered into IBM SPSS (IBM SPSS Corporation, version 28; Rochester, NY, USA), and a two-sided *P*-value of <0.05 was accepted as a suitable level of significance. Descriptive statistics are presented as proportions or mean ± standard deviation as appropriate. Emissions outcomes are reported as median [interquartile range (IQR)] and groups were compared using the Wilcoxon signed-rank Test. When comparing participants with and without SSI, Mann–Whitney *U* test was used to assess significance across groups. Calculations for carbon offsetting value in trees planted are based upon the kgCO_2_e sequestered by a 10-year-old, 5-m tall, 25-cm diameter tree with dry weight of 155.6 kg (25).

## Results

A total of 57 patients were eligible to be included, with 31 agreeing to participate (54.4%). Table 1 outlines baseline characteristics of the included participants. At day 30 follow-up, 28 patients had completed remote follow-up. There were two (6.5%) perioperative mortalities due to ischaemic heart disease and irretrievable limb ischaemia. One (3.2%) participant developed surgical site infection postoperatively requiring significant re-intervention. This resulted in a prolonged admission; hence, the 30-day follow-up was conducted on the ward. This patient was excluded, leaving 28 patients within this analysis. At follow-up, 8 of the 28 participants had developed SSI, giving an infection rate of 28.6%. One patient required readmission for further investigation but not surgical intervention. The remote assessment method correctly identified 7 participants with SSI and 18 participants without SSI. The sensitivity and specificity for identifying SSI were 87.5% and 90.0%, respectively. Using a remote assessment approach resulted in a mean reduction in review time of 12.8 ± 7.5 min per patient (Clinic vs. Remote; 16.6 ± 7.6 vs. 3.8 ± 0.3).

**Table 1:** Baseline characteristics of participants.

	Participants (*n* = 31)	SSI (*n* = 9)	No SSI (*n* = 22)
Sex			
Male	22 (71.0)	5	17
Female	9 (29.0)	4	5
Age (years)			
Mean (SD)	66.7 (11.7)	65.6 (13.8)	67.2 (11.0)
Ethnicity			
White	31 (100.0)	9 (29.0)	22 (100.0)
BMI			
Obese	5 (16.1)	3 (9.2)	2 (6.5)
Not obese	26 (83.9)	7 (22.6)	20 (64.5)
Smoking status			
Smoker	11 (35.5)	5 (16.1)	6 (19.4)
Ex-smoker	14 (45.2)	3 (9.7)	11 (35.5)
Non-smoker	6 (19.4)	1 (3.2)	5 (16.1)
Diabetes			
Insulin dependent	4 (12.9)	2 (6.5)	2 (6.5)
Non-insulin dependent	9 (29.0)	1 (3.2)	8 (25.8)
None	18 (58.1)	6 (19.4)	12 (38.7)
CVA			
Yes	2 (6.5)	0 (0.0)	2 (6.5)
No	29 (93.5)	9 (23.0)	20 (64.5)
Hypertension			
None	10 (32.3)	3 (9.7)	7 (22.6)
No medication	6 (19.4)	2 (6.5)	4 (12.9)
One agent	5 (16.1)	1 (3.2)	4 (12.9)
Two agents	7 (22.6)	1 (3.2)	6 (19.4)
Three or more agents	3 (9.7)	2 (6.5)	1 (3.2)
Peripheral vascular disease			
Yes	31 (100.0)	9 (29.0)	22 (71)
Respiratory disease			
Yes	9 (29.0)	4 (12.9)	5 (16.1)
No	22 (71.0)	5 (16.1)	17 (54.8)
Renal disease			
Yes	4 (12.9)	2 (6.5)	2 (6.5)
No	27 (87.1)	7 (22.6)	20 (64.5)
Immunosuppressants			
Yes	1 (3.2)	1 (3.2)	0 (0.0)
No	30 97.8)	8 (25.8)	22 (71.0)
Baseline creatinine			
Umol/L	83.5 (27.7)	86 (27.3)	82.5 (28.5)
Index procedure			
Common femoral endarterectomy	9 (29.0)	4 (12.9)	5 (16.1)
Femoral-distal bypass	7 (22.6)	2 (6.5)	5 (16.1)
Femoral-popliteal bypass	11 (35.5)	2 (6.5)	9 (23.0)
Femoral-femoral bypass	1 (3.2)	0 (0.0)	1 (3.2)
Aorto-bifemoral bypass	2 (6.5)	1 (3.2)	1 (3.2)
Below knee amputation	1 (3.2)	0 (0.0)	1 (3.2)

CVA, cerebrovascular accident.

The median (IQR) reduction in carbon emissions of remote compared with clinic follow-up was 41.2 (24.5–80.3) kgCO_2_e (*P* < 0.001). The carbon offsetting value using remote-first follow-up is planting one tree for every 6.9 patients. Total carbon footprint of face-to-face follow-up was 2,895.3 kgCO_2_e, compared with 1,301.3 kgCO_2_e when using a remote-first approach (*P* < 0.001), providing an offsetting value of planting 5.6 trees.

Median and total emissions values for participants with SSI are provided in Table 2, in addition to healthcare resource use. Of those who had a diagnosis of SSI (eight), most (five of eight, 62.5%) had 7 days of antibiotics, half (four of eight, 50.0%) had three additional healthcare visits with equal numbers receiving one and two additional visits (two of eight, 25.0%). One participant (12.5%) required readmission for intravenous antibiotics, and subsequently incurred 13 additional bed days. In the 20 participants without wound complications, utilising a remote-first approach improved the environmental impact of follow-up. Median (IQR) reduction in emissions for participants without infection reviewed by remote compared with clinic models were 32.4 (24.4–43.9) kgCO_2_e (*P* < 0.001). Total carbon footprint without wound complications was 700.2 kgCO_2_e for the clinic method and 28.8 kgCO_2_e for remote follow-up.

**Table 2:** Healthcare resource use for participants with SSI.

Patient	GP face-to-face	GP remote	Clinic face-to-face	DN F2F	Nurse clinic face-to-face	Antibiotics	Antibiotic days	Blood bottles	MCS swabs	CT imaging	USS imaging	Hospital bed days	Re-intervention	kgCO_2_e by postoperative remote follow-up	kgCO_2_e by face-to-face follow-up	*P*-value
1	1	0	0	2	0	Flucloxacillin	14	0	1	1	1	0	0	20.43	192.92	
2	1	0	0	0	1	Flucloxacillin	7	0	0	0	0	0	0	2.12	114.91	
3	0	1	0	0	0	Flucloxacillin	7	0	0	0	0	0	0	3.40	187.85	
4	0	1	0	2	0	Flucloxacillin/Co-trimoxazole	14	20	2	1	0	13	0	1,183.76	1,219.93	
5	1	0	0	2	0	Flucloxacillin	14	0	0	0	0	0	0	20.22	126.03	
6	0	0	1	2	0	Co-amoxiclav	7	0	0	0	0	0	0	20.77	126.52	
7	1	0	0	0	0	Flucloxacillin	7	0	0	0	0	0	0	19.67	92.96	
8	0	0	0	2	0	Flucloxacillin	7	0	0	0	0	0	0	2.12	124.08	
Median kgCO_2_e (IQR)														19.95 (3.1–20.5)	129.16 (125.6–190.0)	0.012
Total kgCO_2_e by follow-up method														1,272.50	2,195.0	0.012

GP, general practitioner; MCS, microcopy culture and sensitivity; USS, ultrasound sonography.

Median and total kgCO_2_e are presented for both remote and face-to-face groups.

Using a clinic approach would have incurred a total of 1,424.3 patient return km travelled. Subsequently, this would result in 300.9 kgCO_2_e, with a carbon offsetting value of 1.1 trees. Median (IQR) distance travelled per patient was 42.5 (7.2–58.7) km.

## Discussion

This pilot study outlines the successful implementation of a prospective hybrid accounting method to model the carbon footprint of healthcare activity. To the authors’ knowledge, it is the first study to prospectively model NHS Carbon Footprint Plus emissions in a comparative cohort, assessing two potential environmental interventions. These results may provide a reference case for further prospective environmental analysis. The “remote-first” postoperative follow-up appears to reduce the carbon footprint in this surgical tertiary centre by 41.2 (24.5–80.3) kgCO_2_e per patient. Widespread deployment of a “telemedicine-first” approach to postoperative follow-up could potentially reduce national surgical emissions in line with the NHS long-term plan and Net-Zero 2045 initiatives (16, 26). Extrapolating data presented here to UK Health Security Agency surveillance reports would provide an annual reduction of 4,524.30 mtCO_2_e, with a carbon offsetting value of 15 trees planted or return flights from London, UK, to Perth, Australia (19, 27). Extrapolating data presented here to UK Health Security Agency surveillance reports would provide an annual reduction of 4,524.30 mtCO_2_e, with a carbon offsetting value of 15,900 trees planted or 2,100 return flights from London, UK, to Perth, Australia (19, 27).

Implementing routine remote-first follow-up is safe and accurate for detecting postoperative wound complications (9). This pilot study highlights the feasibility of employing simple measures to achieve asynchronous data collection, although an effective user-friendly interface has been utilised elsewhere (28). The Department of Health and Social Care Medical Technology Strategy and Royal College of Surgeons guidance outline the significance of adopting efficient models of care and improving patient outcomes through early detection (29, 30). In the wake of the SARS-CoV-2 pandemic, innovative strategies are required to streamline surgical care services that can be achieved through remote postoperative follow-up.

SSI rates captured in this study are high (28.6%), but comparative to other literature involving vascular groin incisions (31, 32). The mean age of participants was 66.7 years, reflecting good engagement with elderly population. Previous studies have included younger participants in postoperative telemedicine studies, which may have reflected age-related usability (3). Interestingly, readmission with SSI without any surgical intervention resulted in emissions of 1,219.93 kgCO_2_e, substantially higher than SSI managed in the community (137.90 kgCO_2_e if clinic review and 12.68 kgCO_2_e if reviewed remotely). The small sample of infections here warrants further investigation into the beneficial environmental impact of preventing SSI.

This study does have some limitations. The hybrid accounting methodology has been proposed as the optimum strategy to achieve accuracy, precision, and cost efficiency in carbon footprint modelling (33), and has been successfully employed previously (10). Telemedicine has also been the focus of a recent retrospective life-cycle assessment; however, prospective assessment enables greater granularity of process analysis within the hybrid approach suggested here (34). While utilising this method enables flexibility in bottom-up and top-down approaches to study design, numerous sources are required to comprehensively cover the emissions factors outlined within and out of scopes 1–3 in the GHG protocol (15–24). A carbon accountant was not utilised in this study, although future projects may consider this addition to augment study methodology. However, systematic processes were followed to map carbon emissions within this study, although specific factors continue to be challenging to quantify, such as room kilowatt hour heating and cubic metre water use. To date, there are no universally agreed upon outcome metrics for carbon footprint analysis. Several outcomes for GHG emissions have been outlined dependent on the project objectives, such as emissions intensity, weighted average carbon intensity, absolute emissions among others, although these are not emphasised in a healthcare context (35). There is significant need for the development and regular updates of core outcome sets and checklists in ensuring comparable and rigorous methodological design of environmental studies in healthcare settings.

Carbon offsetting was presented here in number of trees planted, although an alternative approach would be to standardise this value per patient. For reference, remote clinics would have an overall offsetting value of 0.13 trees/patient with the figures here as follows: 0.37 trees/patient for those with infection and 0.11 trees/patient without SSI. While projections based upon national registry data are proposed, the sample size within this pilot study is small, limiting the generalisability of findings. Further studies in pan-specialty postoperative clinics are needed to corroborate these models. For holistic assessment of the environmental impact, all postoperative infections should be mapped with full cost analysis, both of which were beyond the scope of this study.

As a pilot environmental modelling study, this methodology has shown to be achievable and reproducible. It provides a possible reference case to base prospective comparisons of environmental interventions on, which may become key outcomes within future trial methodology alongside cost utility analyses. In addition, it adds key data to the growing body of evidence supporting the benefits of remote postoperative follow-up. A larger cohort will follow this pilot, aligning monetary values with carbon footprint outcomes to further quantify the benefits evidenced here.

## Data Availability

The raw data supporting the conclusions of this article will be made available by the authors, without undue reservation.
